# Accelerated Solvent Extraction as an Alternative for the Recovery of Phenolic Compounds from Chestnut Bur: Optimization of Extraction Conditions

**DOI:** 10.3390/antiox15020207

**Published:** 2026-02-04

**Authors:** Ana I. Paniagua-García, Lucía Gómez-González, Silvia González-Rojo, Rebeca Díez-Antolínez

**Affiliations:** 1 Centre of Biofuels and Bioproducts, Agricultural Technological Institute of Castilla y León, Villarejo de Órbigo, 24358 León, Spaindieantre@itacyl.es (R.D.-A.); 2 Department of Applied Chemistry and Physics, University of León, Campus de Vegazana s/n, 24071 León, Spain; sgonr@unileon.es

**Keywords:** accelerated solvent extraction, chestnut bur, optimization, phenolic compounds, gallic acid

## Abstract

Chestnut bur (CB) is a solid waste product generated in large quantities during the harvesting of edible fruits. This by-product is rich in total phenolic content (TPC) with high antioxidant properties, making it suitable for use in a variety of industrial applications. In this study, the operational variables of accelerated solvent extraction (ASE) and conventional solvent extraction (CSE) of CB were optimized in order to obtain extracts with maximum levels of TPC. The analysis revealed that the extract obtained by ASE using 31.3% ethanol at 180 °C for 9 min achieved the highest value of TPC (8.37 ± 0.05 g gallic acid equivalents (GAE)/100 g dry matter (DM)). Moreover, this extract exhibited higher values of radical-scavenging activity for α,α-diphenyl-β-picrylhydrazyl (DPPH) (90.8 ± 0.3%) than those observed for catechin standard (88.7 ± 0.2%). In addition, its phenolic composition revealed high amounts of gallic acid (13.22 ± 1.01 mg/g DM), followed by 3,4-dihydroxybenzoic acid (2.96 ± 0.16 mg/g DM). This study demonstrates the potential for valorization of CB by ASE under feasible extraction conditions, thereby promoting the circular economy.

## 1. Introduction

In recent years, there has been a significant increase in consumer demand for natural bioactive compounds as alternatives to synthetic chemical products, which are often associated with adverse health effects on humans, animals and the environment [1]. As a result, the circular economy has developed considerably, allowing the use of a wide range of natural waste materials, including those derived from food, industry and agriculture/forestry, for the production of products for various applications [2]. In this context, natural extracts from agri-food wastes, such as chestnut by-products, obtained using environmentally friendly technologies, are considered an important source of biocompounds with potential applications in pharmaceutical, nutraceutical and cosmetic formulations [3].

The European chestnut (*Castanea sativa* Mill.) is a member of the beech family (*Fagaceae*) and represents an important chestnut cultivar produced in temperate regions, including the Mediterranean [4]. In 2022, approximately 308,000 tons of chestnuts were produced in southern Europe, with Spain contributing almost 55% of the total European chestnut production [5]. The processing of whole chestnuts results in significant amounts of waste, including leaves, burs and shells, representing approximately 15% of total production. This poses significant challenges in terms of disposal and environmental impact [6]. In the case of chestnut burs (CB), this waste is typically left in the field after the harvesting season, which can encourage the growth of insect larvae that can ultimately lead to crop damage [7]. To prevent this, some farmers burn the CB, which has a negative impact on the environment. Nevertheless, this by-product is a rich source of phenolic compounds such as gallic acid, flavonoids and tannins, which have antioxidant, antimicrobial, pharmaceutical and nutraceutical properties [6].

A variety of extraction techniques have been used by industry to recover and concentrate the phenolic compounds present in natural matrices. Conventional solvent extraction (CSE) is a relatively simple technique in which the sample is brought into contact with the solvent at the desired temperature and under agitation, using a heating plate or a Soxhlet extractor [8]. However, CSE has several drawbacks, including long extraction times, high energy consumption, low extraction efficiency, the use of large amounts of solvent and the potential thermal degradation of compounds [8,9]. As an alternative, green techniques, including supercritical CO_2_ extraction (sc-CO_2_), microwave-assisted extraction (MAE), ultrasonic-assisted extraction (UAE) and accelerated solvent extraction (ASE), have been proposed as efficient and economically attractive options for industrial processes [8]. In this way, ASE, also known as Pressurized Liquid Extraction (PLE), is considered a green technique that can be efficiently used to recover high-value compounds from food matrices and by-products [10]. Compared to CSE, ASE has the advantage of reducing the volume of solvent required while increasing the extraction rate due to the use of temperatures above the solvent boiling point and the application of high pressures to keep the solvents in a liquid state [11]. In addition, the commercial equipment used for ASE can be programmed and operated in an automated manner, improving reproducibility and quality control and may have favorable characteristics for scaling up the valorization of natural resources [12]. However, ASE has certain limitations, including low selectivity, dilution of recovered compounds, especially when extraction is performed in successive cycles, and high equipment cost. Nevertheless, the exceptional capabilities of ASE can offset the initial investment in a relatively short period of time [12].

In addition to the selection of the extraction technique, the choice of solvent is a critical factor influencing the yield and characteristics of the bioactive compounds recovered. In order to develop processes that are more environmentally friendly, it would be preferable to use non-toxic and renewable solvents, such as water and ethanol [13]. Furthermore, other variables, such as temperature and extraction time, are also important factors in the recovery of phenolic compounds. Therefore, for each type of material and extraction technique, it is crucial to optimize the extraction variables in order to maximize the recovery of phenolic compounds. In this context, several studies have been carried out with the aim of optimizing the extraction variables of phenolics from chestnut by-products. These include studies conducted on chestnut shells by autohydrolysis [8]; MAE with water [14]; MAE with a NaOH solution [15]; ultrasound-assisted deep eutectic solvent extraction [16]; chestnut bark using subcritical water [17]; and CB using CSE with water or hydroalcoholic mixtures [18,19]. In addition, some studies have documented the extraction of phenolic compounds from CB using green extraction techniques, including ethanol-modified sc-CO_2_, autohydrolysis, MAE and UAE [3,20,21,22]. Although there have been previous studies on phenolic extraction from various by-products, including strawberry leaves, saffron processing waste, grape skin and *Thymus serpyllum* herbal dust, using ASE or (PLE) [23,24,25,26], the only documented instance of such extraction from chestnut by-products is found in the study performed by Pazara et al. [27]. In their research, Pazara et al. [27] optimized the PLE variables to maximize the recovery of phenolic compounds from chestnut shell, and they observed that the extract obtained under optimal conditions (40% *v*/*v* of ethanol in the hydroalcoholic mixture at a temperature of 160 °C for 25 min) achieved a TPC recovery that was approximately 500% higher than that achieved in the CSE extract obtained by stirring under the same conditions in terms of liquid-to-solid ratio, solvent and extraction time. Apart from the extraction technique, the only difference in producing the chestnut shell extracts was the extraction temperature, which was 40 °C in CSE. Therefore, the ASE effectiveness in recovering bioactive compounds from agri-food by-products, such as chestnut shells, has been demonstrated. In this context, it is important to note that ASE has not yet been investigated as a potential alternative to valorize CB, with the aim of reducing its environmental impact and promoting a circular economy.

This research presents an experimental evaluation of the effects of the operational variables of CSE and ASE on the phenolic extraction from CB using food-grade solvents. To the best of our knowledge, no studies based on the phenolic extraction from CB using ASE have been found in the literature. The objectives of this work were: (i) to optimize the conditions for CSE and ASE using water and hydroalcoholic mixtures (solvent composition, temperature and extraction time) to maximize the recovery of phenolic compounds, (ii) to verify the effectiveness of the optimal extraction conditions on CB and to compare the results obtained with both extraction techniques and (iii) to determine the chemical composition and evaluate the 2,2-diphenyl-1-picrylhydrazine (DPPH) and ferric reducing antioxidant power (FRAP) assays of the optimal extracts obtained from CB under optimal conditions.

## 2. Materials and Methods

### 2.1. Materials

All chemicals and solvents used were of analytical grade and were obtained from commercial sources. The following are the analytical standards: gallic acid monohydrate, 3,4-dihydroxybenzoic acid, 2,5-dihydroxybenzoic acid, 4-hydroxybenzoic acid, 3-hydroxybenzoic acid, vanillic acid, caffeic acid, syringic acid, vanillin, *p*-coumaric acid, syringaldehyde, ferulic acid, (+)-catechin hydrate, and ascorbic acid. These were purchased from Sigma-Aldrich (Steinheim, Germany). Deionized water (resistivity > 18 MΩ/cm) was prepared using a Milli-Q ultrapure system from Millipore (Burlington, MA, USA).

CB, cultivar Parede, was provided by Fundación Cesefor (Soria, Spain) and collected in November 2021. This by-product was dried in an oven at 45 °C for 48 h, ground in a rotary mill SM100 Comfort (Retsch GmbH, Haan, Germany), sieved to a size of 0.5–1.0 mm (Retsch), and stored at room temperature in airtight containers until use. The compositional analysis of the CB (dry basis) was: 22.44 ± 0.29% cellulose, 17.01 ± 0.31% hemicellulose, 17.76 ± 0.41% Klason lignin, 5.15 ± 0.25% protein, 1.64 ± 0.05 fat and 4.02 ± 0.06% ash. It is important to note that the total monomeric sugar content was 44.25 ± 0.62%. The chemical characterization was carried out according to Paniagua-García et al. [28].

### 2.2. Phenolic Compounds Extraction from CB

#### 2.2.1. Conventional Solvent Extraction

The CSE was performed on 1 g of crushed CB in 100 mL Erlenmeyer flasks and extracted with 50 mL of solvent. The extraction solvent used for each experiment was determined by the experimental design and was composed of water, ethanol, or a mixture of both solvents in various ratios (Section 2.3.1). The flasks were capped with a rubber septum and subjected to magnetic stirring (200 rpm) at the designated temperature and for the specified time, as outlined in Section 2.3.1 of the experimental design. Subsequent to the solid–liquid (S-L) extraction, the mixture was allowed to cool, after which the liquid extract was recovered by vacuum filtration using a Büchner funnel with cellulose filters (20–25 µm, model 1238, Filter Lab, Barcelona, Spain). The spent solids retained in the cellulose filters were washed at room temperature with two aliquots of 20 mL of the fresh extraction solvent used for each experiment and were collected by vacuum filtration. Then, the washing liquid was added to the liquid extract. All extracts were subsequently diluted to a volume of 100 mL and stored at −20 °C until analysis.

#### 2.2.2. Accelerated Solvent Extraction

The ASE was performed using a Dionex ASE 350 (Thermo Fisher Scientific, Waltham, MA, USA) with a solvent controller to maintain the desired solvent ratios. Briefly, 1 g of crushed CB was mixed with 2 g of diatomaceous earth and packed into a 34 mL stainless-steel extraction cell. The cells were equipped with a stainless-steel frit and a cellulose filter (Thermo Fisher Scientific) at the bottom to prevent the collection of suspended particles in the vial. Depending on the experimental design, different solvents (water, ethanol, methanol, ethanol–water and methanol–water mixtures) were tested along with extraction temperatures and times (Section 2.3.2). The first step in the extraction process involved filling the cell containing the sample with the extraction solvent and subsequently pressurizing it to 1500 psi (10.3 MPa). The temperature was then adjusted to the desired extraction temperature, and the static time was set to 0 min. The static phase of the extraction was carried out with all valves closed for the specified extraction time while maintaining a constant pressure and temperature. Following the static extraction process, the cell and tubing were rinsed with 60% of the cell volume of fresh extraction solvent (flush volume). Then the cell was purged with nitrogen for 90 s to remove any residual solvent. The extraction process was executed through a single static cycle. The resulting extracts were collected in a bottle, and the unit was depressurized. To prevent carryover from one experiment to the next, the system was thoroughly rinsed between successive extractions. The resulting extracts were carefully removed from the collection vessel, diluted to a precise volume of 100 mL and stored at −20 °C until analysis.

### 2.3. Experimental Designs and Optimization of Phenolic Extraction Conditions

The operational variables of CSE and ASE of CB were optimized using Response Surface Methodology (RSM) to maximize the extraction efficiency of phenolic compounds.

#### 2.3.1. Optimization of the Recovery of Phenolic Compounds by CSE

Two central composite rotatable designs (CCRDs) were used to optimize phenolic extraction by CSE from CB: one for water-based solvent extraction and the other for the use of a water–ethanol mixture. For the water extraction, a two-variable CCRD, resulting in 13 trials, was used to determine the optimal combinations of temperature (°C) and extraction time (h) to obtain maximum phenolic recovery. The temperature ranged from 50 to 95 °C, the stirring time ranged from 0.50 to 2.50 h, and the stirring speed was maintained at 200 rpm. For the extraction performed with a water–ethanol mixture, a three-variable CCRD, resulting in 20 trials, was designed to calculate the optimal combinations of temperature (°C), stirring time (h) and ethanol concentration (%, *v*/*v*) to maximize phenolic extraction. In this case, the temperature ranged from 40 to 80 °C, the stirring time from 0.50 to 2.50 h, the percentage of ethanol from 0 to 100%, and the stirring speed was maintained at 200 rpm for all the experiments. Each trial extraction was performed in a single replicate.

#### 2.3.2. Optimization of the Recovery of Phenolic Compounds by ASE

Three CCRDs were used to optimize the operating variables of the ASE to maximize the recovery of phenolic compounds from CB. Therefore, for water-based ASE, a two-variable CCRD (13 trials) was used to determine the optimal combinations of temperature (°C) and heating time (min). The temperature was adjusted between 50 and 180 °C, and the heating time was set between 1 and 30 min. In addition, two three-variable CCRDs (each with 20 trials) were designed for the variable optimization of ASE carried out with hydroalcoholic mixtures (one with water–ethanol mixtures and the other with water–methanol mixtures). For both experimental designs, the temperature ranged from 50 to 180 °C, the stirring time from 1 to 30 min and the alcohol concentration from 0 to 100%. Each experiment was performed in a single replicate.

For the five experimental CCRDs, the output results, total phenolic content (TPC), were fitted to second-order polynomial equations. Regression coefficients were determined using analysis of variance (ANOVA), and optimal extraction variables were calculated from each one of the five mathematical models. To validate the regression models, further CSE and ASE experiments were performed under the predicted optimum conditions for each type of extraction and solvent (water, ethanol–water or methanol–water).

### 2.4. Characterization of the Extracts

#### 2.4.1. Total Phenolic Content

The TPC of the CB extracts was determined spectrophotometrically using the Folin–Ciocalteu reagent (Sentmenat, Spain) according to the procedure described by Paniagua-García et al. [1], with some modifications. Briefly, an aliquot of 50 µL of each extract was mixed with 335 µL of Folin–Ciocalteu reagent and diluted with 1.7 mL of deionized water. After resting in the dark for 3 min, 2.5 mL of 7.5% (*w*/*v*) Na_2_CO_3_ solution was added. The reaction mixture was incubated in the dark for 60 min. The absorbance was measured at 740 nm against a blank without phenolic extract using a Cary 50 spectrophotometer (Agilent Technologies, Santa Clara, CA, USA). The results were expressed as g gallic acid equivalents (GAE)/100 g dry matter (DM). All the analyses were conducted in triplicate, and the final results were the average of these.

#### 2.4.2. Total Flavonoid Content

The total flavonoid content (TFC) of the CB extracts was determined using the method described by Squillaci et al. [4], with some modifications. Briefly, 500 µL of phenolic extract was mixed with 1.25 mL of deionized water and 75 µL of 5% (*w*/*v*) NaNO_2_ solution. The resulting solution was left in the dark for 5 min, then 150 µL of 16% (*w*/*v*) AlCl_3_·6H_2_O solution was added. After 1 min, 500 µL of 1 mol/L NaOH solution and 250 µL of deionized water were added. The mixture was shaken vigorously, and the absorbance was measured at 510 nm with a Cary 50 spectrophotometer against a blank containing 500 µL of deionized water. The results were expressed as g catechin equivalents (CE)/100 g DM. The analyses were performed in triplicate, and the results evaluated comprise their average.

#### 2.4.3. Tannin Content

The total tannin content (TTC), total hydrolyzable tannin content (THTC), and the total proanthocyanidin content (TPrC) of the CB extracts were analyzed using the method described by Squillaci et al. [4], with some modifications. For the determination of TTC, 800 µL of phenolic extract was added to 800 µL of 0.5% (*w*/*v*) cinchonine hemisulfate. The resulting solution was vortexed and incubated overnight at 4 °C to achieve quantitative tannin precipitation. After a previous centrifugation at 14,000× *g* for 5 min at 4 °C, the precipitate contained the TTC, while the supernatant contained the remaining phenolics. The non-tannin content was estimated by analyzing the phenolic content of the supernatant according to the Folin–Ciocalteu method. The TTC was calculated as the difference between the TPC of the initial CB extract and the phenolic content of the non-tannin supernatant.

For quantification of THTC and TPrC, the tannin-containing precipitate was dissolved in 800 µL of ethanol–water (1:1). Then, 500 µL of the tannin solution was added to 250 µL of HCl–water (2:5) and 250 µL of 4.8% (*v*/*v*) formaldehyde. The mixture was shaken vigorously, left overnight at room temperature and centrifuged at 14,000× *g* for 30 min at 4 °C. The phenolic content of the supernatant was measured using the Folin–Ciocalteu assay, and the result represented the THTC. The TPrC (precipitate fraction) was calculated as the difference between the TTC and the THTC. The results of tannin content were expressed as g GAE/100 g DM. All the analyses were conducted in triplicate, and the final results were the average of these.

#### 2.4.4. HPLC Analysis of Phenolic Compounds

The concentration of the following individual phenolic compounds was determined in the optimal CB extracts after hydrolysis of the gallotannins with HCl (final concentration 2.5 M) for 1 h at 90 °C [29]: gallic, 3,4-dihydroxybenzoic, 2,5-dihydroxybenzoic, 4-hydroxybenzoic, 3-hydroxybenzoic, vanillic, caffeic, syringic, *p*-coumaric, and ferulic acids, as well as vanillin and syringaldehyde. Subsequently, the aforementioned compounds were analyzed using an Agilent 1260 Infinity II Prime LC system (Agilent Technologies, Santa Clara, CA, USA) equipped with a diode array detector (DAD) and a Waters Resolve C18 analytical column (300 mm × 3.9 mm, 5 µm) (Waters Corporation, Milford, MA, USA), according to the analytical method described by Paniagua-García et al. [28]. In summary, the mobile phase comprised two solvents: (A) an aqueous solution of 1% (*v*/*v*) acetic acid, with the pH adjusted to 2.5 by the addition of H_3_PO_4_, and (B) acetonitrile. The flow rate was set at 0.9 mL/min with the following gradient program: 95% (A) isocratic (15 min), 95–70% (A) (13 min), 70–95% (A) (2 min), with a post-run of 5 min. The chromatographic column was maintained at 35 °C. The injection volume was 20 µL, and the detection was carried out at four different wavelengths: 235, 254, 276 and 320 nm. Extract samples were filtered through a 0.22 µm nylon syringe filter (Agilent Technologies) prior to injection. The analyses were performed in triplicate, and the results were the average of them expressed as mg/g DM.

#### 2.4.5. Antioxidant Activity

##### Ferric Reducing Antioxidant Power

The ferric reducing antioxidant power (FRAP) of CB extracts was evaluated using a modified version of the method described by Chiancone et al. [30]. Briefly, the FRAP reagent solution was prepared by mixing 100 mL of sodium acetate buffer solution (pH 3.6), 10 mL of 10 mmol/L 2,4,6-tris(2-pyridyl)-s-triazine (TPTZ) solution in 40 mmol/L HCl and 10 mL of 20 mmol/L FeCl_3_·6H_2_O. Then, 100 µL of phenolic extract or ascorbic acid standard solution was mixed with 3.0 mL of the FRAP reagent. The solution was preincubated for 5 min in the dark at room temperature. The absorbance of the standards and samples was then measured at 593 nm using a Cary 50 spectrophotometer against a blank containing 100 µL of deionized water. The results were expressed as g ascorbic acid equivalents (AAE)/100 g DM. The analyses were conducted in triplicate, and the final results were the average of these.

##### DPPH Free Radical-Scavenging Activity

The antiradical capacity of CB extracts was determined using the 2,2-diphenyl-1-picrylhydrazyl (DPPH) assay according to Squillaci et al. [4], with some modifications. Briefly, the extract was diluted with water to different concentrations ranging from 1.5 to 90 µg GAE/mL. Next, 300 µL of the diluted sample was mixed with 2.7 mL of a methanolic solution containing 60 µmol/L DPPH radicals. The mixture was vortexed and allowed to stand in the dark for 30 min. DPPH radical reduction was then measured by reading the absorbance at 517 nm against a control solution containing 300 µL of deionized water. The radical-scavenging activity (RSA) was calculated as follows: RSA (%) = 100 × [(A_0_ − A_S_)/A_0_], where A_0_ is the absorbance of the control solution, and A_S_ is the absorbance of the diluted sample. The concentration of the extract that provides the maximum radical-scavenging activity (CRSA_max_) and the concentration of the extract that provides 50% of the radical-scavenging activity (IC50) were calculated by plotting RSA versus extract concentration. The results were expressed as mg CB/mL required to achieve CRSA_max_ or IC50, respectively. All the analyses were performed in triplicate, and the results evaluated comprise their average. Catechin and ascorbic acid (reference antioxidant compounds) were used as standards.

### 2.5. Statistical Analysis

The experimental designs were analyzed using the RSM based on CCRD using Minitab 16 software (Minitab, State College, PA, USA). Second-order polynomial models were fitted to describe the relationship between the independent variables (temperature, extraction time, and, where applicable, ethanol or methanol concentration) and the response variable (TPC) for each CCRD.

The adequacy of each model was evaluated using ANOVA to assess overall model significance (*p* value < 0.05) and the R^2^ to determine the goodness of fit. The residual plots were examined to verify the normality assumptions (Shapiro–Wilk test) and the homogeneity of variance assumption.

Contour and response surface plots were generated to visualize the main and interaction effects of the independent variables on TPC and to identify the optimum extraction conditions. The predicted optimum values obtained from the models were validated through experimental testing under the predicted conditions. The calculated and observed responses were compared to confirm the validity of the model.

For extracts of CB obtained under the optimized conditions, results were expressed as the mean ± standard deviation (n = 3). Differences among extraction treatments were analyzed by one-way ANOVA, and Tukey’s HSD test was used as a post hoc comparison procedure at a significance threshold of *p* > 0.05 (Statistix 10.0 Analytical Software, Tallahassee, FL, USA).

## 3. Results and Discussion

### 3.1. CSE Optimization

In order to maximize the recovery of phenolics from CB by CSE with water, thirteen experiments were conducted and yielded variable results ranging from 3.22 to 5.71 g GAE/100 g DM (Table 1). In addition, to maximize the phenolic recovery from CB by CSE with a water–ethanol mixture, twenty experiments were performed and showed values ranging from 0.67 to 5.71 g GAE/100 g DM (Table 2).

**Figure 1:**
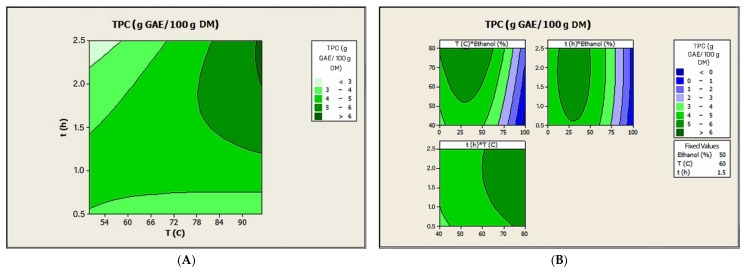
Contour plots between the coupled independent variables of CSE performed with water (**A**) or water–ethanol (**B**) for the recovery of phenolic compounds from CB.

Two ANOVAs were performed to analyze the experimental results and to evaluate the effects of the independent variables and their possible interactions on CSE with respect to the extraction solvent used. The quadratic model explained 86.90% of the variation in phenolic recovery from CB by CSE with water (Table S1). A similar model explained 99.57% of the variation in phenolic recovery using water–ethanol mixtures (Table S2). Although the mathematical model calculated for the CSE performed with water indicated only moderate acceptability, the model can be fully accepted when a water–ethanol mixture is used. Furthermore, the predicted quadratic models for the recovery of phenolics with both extraction solvents are significant at the 95% confidence level with *p*-values less than 0.05 (0.005 and 0.000, respectively) (Tables S3 and S4). Moreover, in the case of water-based CSE, the contour plots confirm that there were significant interactions (*p* < 0.05; Table S3; Figure 1A) between stirring time and temperature. Even though the corresponding interaction terms were not statistically significant (*p* > 0.05; Table S4), the contour plots in Figure 1B show that there were interactions between temperature and ethanol concentration and between stirring time and ethanol concentration on phenolic recovery when a water–ethanol mixture was used. For water-based CSE, stirring time had a greater influence than temperature on the phenolic recovery values. Although the effect of both independent variables on the response variable analyzed was not significant and negative, it should be noted that the lowest results were obtained at the lowest time (0.50 h: 3.25 g GAE/100 g DM) and at the lowest temperature (50 °C: 4.09 g GAE/100 g DM). In contrast, the influence of stirring time on phenolic recovery using a water–ethanol mixture was the highest, followed by the ethanol concentration and temperature. Furthermore, in this case, all variables showed a positive effect, but only the effect of ethanol concentration on phenolic recovery was statistically significant. These results are consistent with those of previous studies, which showed that the main independent variables affecting CSE of CB using water–ethanol mixtures were temperature and solvent concentration, while the influence of time was found to be minimal [18,31].

The optimal values of the independent variables were calculated, and the results showed that the water-based CSE of CB at 95 °C with a stirring time of 1.98 h achieved an estimated phenolic recovery of 6.10 g GAE/100 g DM. In the case of using a water–ethanol mixture, the CSE performed at 80 °C with a stirring time of 2.22 h and 31.3% (*v*/*v*) of ethanol predicts a phenolic recovery of 6.12 g GAE/100 g DM. After experimental validation of the optimal operating conditions for both extraction solvents in triplicate, the phenolic recovery was found to be 6.04 ± 0.14 g GAE/100 g DM using water and 5.70 ± 0.07 g GAE/100 g DM using the water–ethanol mixture. These experimental results confirmed that both calculated models were adequate and valid enough to achieve the optimization results.

While the calculated phenolic recovery in the extracts obtained under optimal conditions was almost identical for both types of solvents, experimental values showed the highest phenolic extraction with water. Despite the presence of phenolic compounds with varying degrees of polarity and solubility in CB, the higher recovery of phenolics by CSE when using water seems to indicate that the phenolic compounds in this matrix exhibit high polarity [27]. Therefore, the experimental results obtained in the present research were in accordance with those previously reported by other researchers who subjected CB to CSE and observed higher values of phenolic extraction with the use of water than with a water–ethanol mixture. Accordingly, Zhao et al. [32] obtained values of 7.12 ± 0.89, 6.56 ± 0.76, 7.00 ± 1.02 and 3.31 ± 0.62 g GAE/100 g DM for phenolic extraction performed with water, 30% ethanol, 50% ethanol and 75% ethanol, respectively, using a water bath with stirring (80 °C, 1 h and solid load of 1/10, *w*/*v*). However, the results obtained by Zhao et al. [32], achieved by applying shorter stirring times and higher solid load, were slightly higher than those obtained in the present study. Furthermore, Vella et al. [31] reported phenolic recovery values from the CSE, with vigorous stirring, of CB of 6.00 ± 0.47 g GAE/100 g DM (boiling water, solid load of 1/40, *w*/*v*) and 5.09 ± 0.33 g GAE/100 g DM (60% ethanol, at room temperature, with a solid load of 1/10, *w*/*v*). Although the results reported by the aforementioned authors are similar to those achieved in the present research, Vella et al. [31] used a shorter stirring time (40 min). Moreover, the phenolic recoveries obtained in the present study were higher than those reported by other authors, such as Fernández-Agulló et al. [22], who achieved amounts of 4.38 and 4.86 g GAE/100 g DM from CB by CSE carried out with water and 50% ethanol, respectively, at 75 °C for 120 min and a solid load of 1/10, *w*/*v*. In addition, Spissu et al. [6] obtainied lower phenolic recovery values from the water-based CSE of CB carried out under continuous stirring in a thermostatic bath, ranging from 3.96 ± 0.17 to 4.30 ± 0.13 g GAE/100 g DM (20 °C, 4 h and solid load of 1/10, *w*/*v*) and from 3.74 ± 0.06 to 4.01 ± 0.22 g GAE/100 g DM (95 °C, 1 h and solid load of 1/20, *w*/*v*).

With regard to the implementation of the RSM approach for the optimization of phenolic recovery from CB through CSE, some works can be found in the literature [18,19]. Consequently, Vázquez et al. [18] achieved a phenolic recovery of 4.69 g GAE/100 g DM from CB using 50% of ethanol in water, a solid load of 1/10 (*w*/*w*), at 75 °C for 30 min with an orbital shaker set at 90 rpm. Furthermore, Gullón et al. [19] obtained a CB extract with a phenolic recovery of 3.39 g GAE/100 g DM in an orbital shaker (120 rpm) with an aqueous ethanol solution (42.5%) and a solid load of 1/20 (*w*/*v*), at 70 °C for 240 min. It is imperative to acknowledge that these variations in phenolic recovery levels might be attributable not only to the conditions of the extraction method but also to the variations in CB composition, which are influenced by factors such as variety, climate, soil, and storage.

### 3.2. ASE Optimization

The operating variables of the ASE were optimized to maximize the phenolic recovery from CB by using three CCRDs: one for the extraction conducted with water, and the other two for the use of two kinds of hydroalcoholic mixtures (ethanol or methanol). In this case, given the lack of studies in the literature based on the ASE of phenolics from CB, the use of a water–methanol mixture was included to plan a third optimization experimental design, because methanol is considered a reference solvent with high extraction capacity, resulting in fractions with high radical scavenging activity [20]. The phenolic recovery ranged from 3.03 to 6.33 g GAE/100 g DM (water-based ASE, Table 3), from 1.66 to 7.44 g GAE/100 g DM (water–ethanol mixture ASE, Table 4), and from 2.46 to 7.67 g GAE/100 g DM (water–methanol mixture ASE, Table 4).

**Figure 2:**
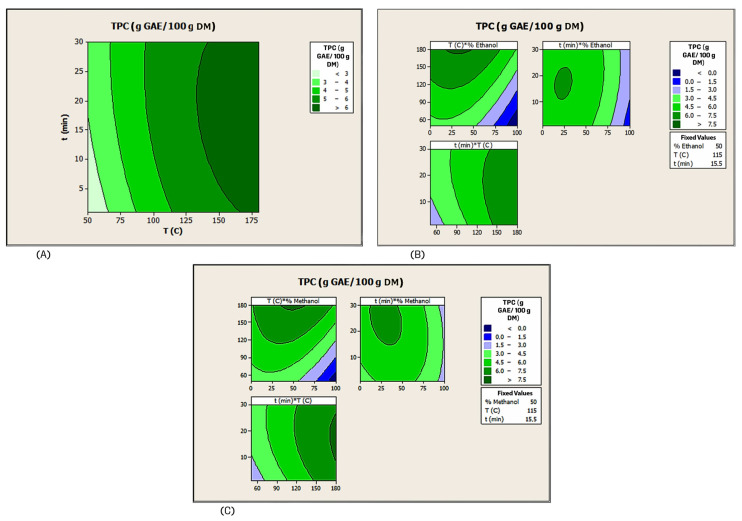
Contour plots between the coupled independent variables of ASE performed with water (**A**), water–ethanol (**B**) or water–methanol (**C**) for the recovery of phenolic compounds from CB.

Three ANOVAs were calculated, one for each experimental design, to analyze the experimental results and to evaluate the effects of the independent variables and their possible interactions on each ASE trial with respect to the extraction solvent used. The calculated quadratic models explained 99.18% of the variation in the ASE of phenolic recovery with water (Table S5), 98.70% with water–ethanol mixtures (Table S6), and 98.19% with water–methanol mixtures (Table S7). Consequently, the three mathematical models showed that they were sufficiently valid and can be fully accepted. Furthermore, the predicted quadratic models for phenolic recovery using the three extraction solvents were significant at the 95% confidence level with *p*-values less than 0.05 (0.000 for the three calculated models) (Tables S8–S10). The contour plots illustrate the combined effects of each pair of independent variables on phenolic recovery by ASE using water, ethanol–water, or methanol–water (Figure 2). Nevertheless, there were no significant interactions (*p* > 0.05; Table S8) between time and temperature on the phenolic recovery when water was used as the extraction solvent. Conversely, using the hydroalcoholic mixtures, there were significant interactions (*p* > 0.05; Tables S9 and S10) between temperature and ethanol concentration and between temperature and methanol concentration, on the phenolic recovery from CB. Regarding the influence of the independent variables on the values of the phenolic recovery, in the case of the water-based ASE, temperature and time have a similar effect, and both variables showed a positive and significant effect. For the ASE carried out with both hydroalcoholic mixtures, temperature and time were significant variables for both mathematical models, and the greatest effect on the phenolic recovery was observed by time, followed by temperature and alcohol concentration.

The optimal values of the independent variables, as well as the estimated phenolic recovery from CB were calculated according to the equations of the mathematical models and were as follows: 173 °C for 17 min, 6.34 g GAE/100 g DM (water-based ASE); 180 °C for 9 min and 31.3% (*v*/*v*) of ethanol, 7.75 g GAE/100 g DM (water–ethanol mixture ASE); 180 °C for 18 min and 48.5% (*v*/*v*) of methanol, 7.70 g GAE/100 g DM (water–methanol mixture ASE). These operating conditions were validated by the extraction of CB in triplicate. The validation process showed phenolic recovery values of 6.90 ± 0.08 g GAE/100 g DM (water-based ASE), 8.37 ± 0.05 g GAE/100 g DM (water–ethanol mixture ASE), and 8.06 ± 0.08 g GAE/100 g DM (water–methanol mixture ASE). Although there were some minor differences between the calculated and experimental values, the results demonstrated the effectiveness and reliability of the three response models in estimating the optimization results.

It is noteworthy that the phenolic recoveries from CB were higher by ASE than by CSE. This phenomenon can be attributed to the enhanced extraction kinetics and yields resulting from the application of temperatures above the solvent boiling point, but bellow critical point, in conjunction with the application of high pressures to maintain the solvents in their liquid state [11]. Under these conditions, the surface tension and viscosity of the solvent are diminished, thereby enhancing its penetration into the solid matrix and facilitating the extraction of compounds located in the internal pores with reduced solvent consumption [33]. Moreover, the findings of the present study on the CSE of CB are contradicted by the results obtained through ASE, which demonstrated that the highest yield of phenolic recovery was achieved with the use of a solvent extraction composed of a water–ethanol mixture. In order to explain this difference, it is necessary to consider that phenolic compounds are typically polar and exhibit a greater hydrophilicity than lipophilicity. Consequently, polar protic solvents generally yield better extraction results. Although water is more polar than ethanol, the high pressure and temperature above its boiling point cause water to become less polar due to the breakdown of intermolecular hydrogen bonds [12]. In hydroalcoholic mixtures, protic ethanol reduces polarity and improves the solubilization of phenolic compounds, while water enhances their desorption from the matrix, which improves the extraction efficiency. Furthermore, the use of co-solvent ethanol has been shown to reduce thermal hydrolysis and degradation of phenolic compounds. These properties of water–ethanol mixtures make them highly suitable for high-pressure and high-temperature techniques, such as ASE [12].

To the best of our knowledge, this is the first time CB has been subjected to ASE to recover bioactive compounds. Moreover, only one study based on the phenolic recovery from chestnut by-products through ASE can be found in the literature [27]. In the aforementioned study, the operational conditions of ASE from chestnut shell were optimized using RSM (Box–Behnken design) to obtain an extract with maximum values of phenolic and tannin recovery and antioxidant properties (FRAP and DPPH antiradical activity). Therefore, the chestnut shell extract obtained with 40% (*v*/*v*) aqueous ethanol at 160 °C for an extraction time of 25 min achieved a phenolic recovery of 11.37 ± 0.78 g GAE/100 g. However, those results could not be compared with those achieved in the present work because the phenolic content of different chestnut by-products is different.

Regarding the application of other “green” extraction techniques to CB, previous studies have reported, with lower phenolic recovery values than those obtained in this study. Therefore, Fernández-Agulló et al. [22] conducted a study on the extraction of phenolic compounds from CB using MAE, setting constant values for solid load and temperature (1/10, *w*/*v* and 15 min). The findings revealed a range of values from 3.19 to 5.77 g GAE/100 g DM, depending on the extraction solvent used (water, 50% methanol, or 50% ethanol) and temperature (50 or 75 °C). Furthermore, Rodrigues et al. [3] reported phenolic extraction values from CB ranging from 0.96 ± 0.01 to 3.69 ± 0.11 g GAE/100 g DM, depending on the extraction technique used (UAE or MAE) and the type of extraction solvent (water or 80% ethanol). Therefore, ASE has been shown to yield a higher recovery of phenolic compounds from CB than other green extraction techniques. This suggests that ASE is a promising technique for recovering phenolic compounds from CB.

### 3.3. Chemical Composition and Antioxidant Activities of the Optimal Extracts

#### 3.3.1. Chemical Composition

As shown in Table 5, the highest values for TPC and TTC were obtained with the water–ethanol mixture ASE (8.37 ± 0.05 and 6.58 ± 0.04 g GAE/100 g DM, respectively) (*p* < 0.05). In the case of TFC and TPrC, the maximum yields were achieved with ASE using a water–ethanol mixture (1.38 ± 0.02 g CE/100 g DM and 4.61 ± 0.02 g GAE/100 g DM, respectively) and a water–methanol mixture (1.32 ± 0.01 g CE/100 g DM and 4.37 ± 0.03 g GAE/100 g DM, respectively), with no significant differences between them (*p* > 0.05). However, in the case of CSE, the phenolic yield was higher when water was used as the extraction solvent. A similar trend was observed for tannin and proanthocyanidin yields. Regarding flavonoid recovery, the use of water or a mixture of water–ethanol in CSE gave equivalent results (*p* > 0.05). For THTC, the highest recovery was obtained for the water–ethanol mixture ASE (1.96 ± 0.03 g GAE/100 g DM), although no statistically significant differences were observed between the values attained for the water-based ASE, the water-based CSE, and the water–ethanol mixture CSE (*p* > 0.05). According to the literature, the predominant hydrolyzable tannins in CB are gallotannins [3,6,34]. The flavonoid recovery values were comparable to those reported by Vella et al. [31], who subjected CB to CSE with vigorous stirring using boiling water for 40 min. However, the tannin recovery in the aforementioned study was lower (0.43 g GAE/100 g DM) than that obtained in the present work. In addition, Spissu et al. [6] obtained comparable results of flavonoid recovery (1.14–1.87 g CE/100 g DM) in CB extracts prepared from a solid load of 1/10 (*w*/*v*), through water-based CSE using a thermostatic bath with continuous stirring at 20 °C for 4 h. Furthermore, Zhao et al. [32] reported lower values of tannin recovery (2.42–5.13 g GAE/100 g DM) in CB extracts obtained by CSE, in a water bath with stirring, using different solvents (water, 30% ethanol, 50% ethanol and 75% ethanol) and a solid load of 1/10, *w*/*v* at 80 °C for 1 h.

**Figure 3:**
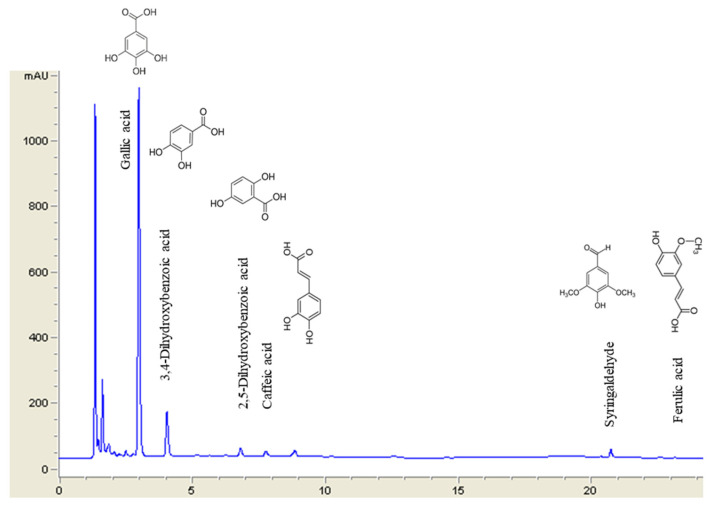
HPLC-DAD chromatogram of the optimal extract of CB obtained by water-based ASE.

In addition, twelve individual phenolic compounds were quantified in the optimal extracts, and slight differences in their values were observed (Table 5). The predominant compound in all extracts was gallic acid (Figure 3), which reached the highest value when CB was subjected to ASE with water or water–ethanol mixture (13.22 ± 1.01 and 12.28 ± 0.16 mg/g, respectively) (*p* < 0.05). Several previous works have reported the presence of gallic acid as the most abundant phenolic compound in CB extracts [3,6,22,35]. Furthermore, the amounts of gallic acid recovered were in agreement with those reported by Rodrigues et al. [3] when CB was subjected to water-based UAE. However, phenolic extraction from CB by hydroalcoholic UAE or water-based MAE resulted in lower gallic acid recovery (7.58 ± 0.01 and 4.28 ± 0.03 mg/g DM, respectively). Moreover, other researchers have reported lower gallic acid extraction from CB by CSE with 70% methanol at 70 °C for 60 min in a screw-top microtube using a vortex mixer every 5 min (0.14 ± 0.04 to 0.36 ± 0.06 mg/g DM) [35] or by water-based CSE with stirring at 20 °C for 4 h (0.13 ± 0.04 to 0.93 ± 0.13 mg/g DM) [6].

Furthermore, 3,4-dihydroxybenzoic acid was the second most abundant compound analyzed, reaching a maximum value in the water-based ASE extract (2.96 ± 0.16 mg/g DM). The maximum values for syringic acid were observed in water–ethanol extracts (1.48 ± 0.31 mg/g with ASE and 1.07 ± 0.24 mg/g with CSE). Other compounds analyzed, including 2,5-dihydroxybenzoic acid, caffeic acid, *p*-coumaric acid, syringaldehyde and ferulic acid, were recovered at levels less than 0.30 mg/g DM. In addition, 4-hydroxybenzoic acid, 3-hydroxybenzoic acid, vanillic acid, and vanillin were not detected (nd) in the CB extracts. These results are in agreement with those previously reported by other researchers, who have identified the presence of 3,4-dihydroxybenzoic acid, syringic acid, *p*-coumaric acid, and ferulic acid in CB extracts [2,13]. Furthermore, Flórez-Fernández et al. [13] documented the presence of 4-hydroxybenzoic acid and vanillic acid. Moreover, other authors have reported the presence of additional compounds not analyzed in the present study in CB extracts, including ellagic acid, castalagin, vestalagin, chestanin, epigallocatechin, quercetin, catechin, and epicatechin [3,6,34,35].

#### 3.3.2. Antioxidant Activities

As expected, the higher TPC of CB extracts resulted in higher FRAP values. Therefore, the maximum FRAP values were obtained in the extracts prepared by ASE, with no significant differences (*p* > 0.05) between the use of the three extraction solvents (5.71 ± 0.16 to 6.38 ± 0.14 g AAE/100 g DM).

**Figure 4:**
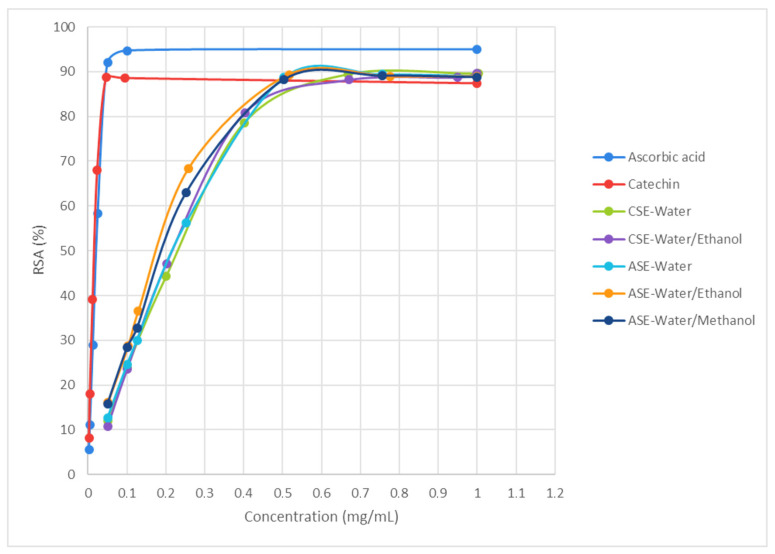
DPPH free radical-scavenging activity (RSA) of CB extracts concentrations obtained by CSE and ASE under optimal conditions and antioxidant reference compounds (ascorbic acid and catechin).

For the DPPH radical-scavenging assay, the RSA values were expressed as the percentage ratio between the decrease in absorbance of the sample and the absorbance of the DPPH solution in the absence of the CB extract at 517 nm. As shown in Figure 4, the scavenging activity of the CB extracts on DPPH radicals shows a concentration-dependent increase, and reaches values comparable to those of ascorbic acid and even exceeds those of catechin. This benefit can be attributed to the additive and synergistic effect of the complex mixture of phytochemicals present in the CB extracts, which are responsible for their robust bioactive properties that exceed those of individual compounds [31]. The extract obtained by water-based ASE produced the highest RSA_max_ value (*p* < 0.05) (91.5 ± 0.1% at 0.54 ± 0.02 mg/mL), followed by those obtained by ASE using water–ethanol and water–methanol mixtures (90.8 ± 0.3% at 0.54 ± 0.02 mg/mL and 90.4 ± 0.3% at 0.54 ± 0.02 mg/mL, respectively). As derived from the highest TPC, the extracts prepared with water–ethanol ASE or water–methanol ASE showed the lowest IC50 values (*p* < 0.05) (0.19 ± 0.01 mg/mL for both solvents). Consequently, these extracts ted exhibited the greatest efficacy in counteracting free radicals. The RSA_max_ and IC50 results obtained in the present study were comparable to those reported by Spissu et al. [6], who studied the production of CB extracts by CSE using hot water at 95 °C under continuous stirring for 1 h. However, although the results of TFC obtained by Spissu et al. [6] were similar to those achieved in the present research, the values of TFC and FRAP reported by those authors were lower (from 3.74 to 4.43 g GAE/100 g DM and from 0.02 to 0.03 g AAE/100 g DM, respectively). In addition, Moure et al. [21] reported higher IC50 values (0.38 g/L) when CB was extracted with absolute ethanol in a Soxhlet apparatus for 8 h than those obtained in this study. Furthermore, Esposito et al. [34] achieved lower values of IC50 (from 0.01 to 0.02 mg/mL) for CB extracts obtained through a sequential extraction performed with the use of several organic solvents (*n*-hexane, chloroform, methanol, *n*-butanol, and aqueous ethanol) than those obtained in the present study.

The results of this research demonstrate that the highest extraction of antioxidant compounds from CB was achieved using water or a water–ethanol mixture. The use of food-grade solvents enhances the industrial relevance and sustainability of the proposed methodologies, aligning with current demands for green and safe extraction processes. In addition, the implementation of alternative extraction techniques, such as ASE, provides new insights into the efficiency of this technique for enhancing phenolic recovery under controlled temperature and pressure conditions. This enables more efficient extraction processes and the potential for achieving higher extraction yields. Furthermore, ASE is a green extraction technique that is easily integrated into the industry [27]. However, the laboratory-scale process has some limitations, and further research is needed to determine how to scale up the process to make it feasible and economically viable.

## 4. Conclusions

This study demonstrates for the first time that extracts with a very high TPC can be recovered from CB by ASE using environmentally friendly solvents and short extraction times. After optimizing the extraction variables, a maximum value of 8.37 ± 0.05 g GAE/100 g DM of TPC was recovered from CB by ASE at 180 °C for 9 min using a hydroalcoholic mixture (31.3% ethanol). Twelve phenolic compounds were analyzed in CB extracts, and gallic acid was identified as the most abundant (13.22 ± 1.01 mg/g DM), followed by 3,4-dihydroxybenzoic acid (2.96 ± 0.16 mg/g DM) and syringic acid (1.48 ± 0.31 mg/g DM). The analysis also revealed that 2,5-dihydroxybenzoic acid, caffeic acid, *p*-coumaric acid, syringaldehyde, and ferulic acid were recovered in amounts below 0.30 mg/g DM. The presence of other phenolics, including 4-hydroxybenzoic acid, 3-hydroxybenzoic acid, vanillic acid, and vanillin, was not detected in the analyzed extracts. The study of the antioxidant properties of CB extracts demonstrated higher values of DPPH radical-scavenging activity (91.5 ± 0.1%) in comparison to those achieved for catechin solutions (88.7 ± 0.2%).

The findings of this study provide a theoretical basis for the development of a valorization process that contributes to the circular economy by converting CB into useful resources. Consequently, the CB extracts obtained through ASE could be used to produce natural antioxidant additives with applications in food, animal feed, and cosmetic industries. However, industrial-scale implementation necessitates further investigation into the scalability and economic feasibility of the process. Moreover, additional research is necessary to ascertain other properties of the CB extracts, including but not limited to antimicrobial properties. Determining these properties will allow for an increased range of applications, thus reducing waste and promoting more efficient use of the materials.

## Figures and Tables

**Table 1:** Experimental design of water-based CSE of CB and the observed results of TPC (g GAE/100 g DM).

Trial	T (°C)	t (h)	TPC (g GAE/100 g DM)
1	57	2.20	3.22
2	57	0.80	4.18
3	88	0.80	4.54
4	88	2.20	5.71
5	73	2.50	4.37
6	73	0.50	3.25
7	73	1.50	4.94
8	73	1.50	4.97
9	73	1.50	4.64
10	73	1.50	4.55
11	73	1.50	4.41
12	95	1.50	5.17
13	50	1.50	4.09

**Table 2:** Experimental design of ethanol–water mixtures CSE of CBs and the observed results of TPC (g GAE/100 g DM).

Trial	Ethanol (%, *v*/*v*)	T (°C)	t (h)	TPC (g GAE/100 g DM)
1	80	48	0.90	2.23
2	80	48	2.10	2.31
3	80	72	2.10	3.53
4	80	72	0.90	3.00
5	50	40	1.50	4.09
6	50	60	1.50	5.02
7	50	60	1.50	5.04
8	50	60	1.50	4.90
9	50	60	1.50	4.82
10	50	60	1.50	4.99
11	50	60	1.50	4.89
12	50	60	0.50	4.53
13	50	60	2.50	4.92
14	50	80	1.50	5.64
15	0	60	1.50	4.28
16	100	60	1.50	0.67
17	20	48	0.90	4.60
18	20	48	2.10	4.88
19	20	72	0.90	5.57
20	20	72	2.10	5.71

**Table 3:** Experimental design of water-based ASE of CB and the observed results of TPC (g GAE/100 g DM).

Trial	T (°C)	t (min)	TPC (g GAE/100 g DM)
1	69	5	3.38
2	69	26	4.01
3	115	30	5.56
4	115	1	4.99
5	115	16	5.51
6	115	16	5.58
7	115	16	5.77
8	115	16	5.51
9	115	16	5.78
10	50	16	3.03
11	180	16	6.19
12	161	5	6.22
13	161	26	6.33

**Table 4:** Experimental designs of hydroalcoholic mixtures (ethanol or methanol) ASE of CB and the observed results of TPC (g GAE/100 g DM).

Trial	Alcohol (%, *v*/*v*) *	T (°C)	t (min)	TPC (g GAE/100 g DM) (Ethanol)	TPC (g GAE/100 g DM) (Methanol)
1	80	76	7	1.89	2.46
2	80	76	24	2.18	2.78
3	80	154	7	4.88	6.26
4	80	154	24	5.27	5.82
5	50	50	16	3.15	3.27
6	50	115	16	5.48	5.80
7	50	115	16	5.62	6.00
8	50	115	16	5.35	5.82
9	50	115	16	5.65	5.93
10	50	115	16	5.61	5.96
11	50	115	16	5.55	5.65
12	50	115	1	4.56	4.55
13	50	115	30	5.62	6.10
14	50	180	16	7.44	7.67
15	0	115	16	5.69	5.42
16	100	115	16	1.66	2.53
17	20	76	7	4.53	4.20
18	20	76	24	5.13	4.95
19	20	154	7	7.30	6.07
20	20	154	24	6.42	6.66

*: Alcohol (ethanol or methanol) percentage in the solvent extraction.

**Table 5:** Chemical composition and antioxidant activities (2,2-diphenyl-1-picrylhydrazyl (DPPH) free radical-scavenging activity and ferric reducing antioxidant power (FRAP)) of CB extracts obtained by CSE or ASE using water, ethanol–water or methanol–water under optimal conditions. Different letters indicate significant differences (Tukey’s HDS; *p* < 0.05) between CB extracts for the same parameter.

	CSE Water	CSE Ethanol/Water	ASE Water	ASE Ethanol/Water	ASE Methanol/Water
**Chemical Composition**					
Optimal conditions	95 °C, 1.98 h	31.3% Ethanol, 80 °C, 2.22 h	173 °C, 17 min	31.3% Ethanol, 180 °C, 9 min	48.5% Methanol, 180 °C, 18 min
Calculated TPC (g GAE/100 g DM)	6.10	6.12	6.34	7.75	7.70
TPC (g GAE/100 g DM)	6.04 ± 0.14 _D_	5.70 ± 0.07 _E_	6.90 ± 0.08 _C_	8.37 ± 0.05 _A_	8.06 ± 0.08 _B_
TFC (g CE/100 g DM)	1.02 ± 0.03 _C_	1.03 ± 0.02 _C_	1.24 ± 0.02 _B_	1.38 ± 0.02 _A_	1.32 ± 0.01 _A_
TTC (g GAE/100 g DM)	5.34 ± 0.15 _C_	4.94 ± 0.06 _D_	5.34 ± 0.07 _C_	6.58 ± 0.04 _A_	6.05 ± 0.14 _B_
TPrC (g GAE/100 g DM)	3.55 ± 0.26 _B_	3.08 ± 0.08 _C_	3.45 ± 0.05 _BC_	4.61 ± 0.02 _A_	4.37 ± 0.03 _A_
THTC (g GAE/100 g DM)	1.79 ± 0.16 _AB_	1.86 ± 0.05 _AB_	1.89 ± 0.08 _AB_	1.96 ± 0.03 _A_	1.68 ± 0.13 _B_
Gallic acid (mg/g DM)	10.40 ± 1.09 _BC_	10.12 ± 1.17 _BC_	13.22 ± 1.01 _A_	12.28 ± 0.16 _AB_	9.49 ± 0.76 _C_
3,4-Dihydroxybenzoic acid (mg/g DM)	0.89 ± 0.06 _B_	0.48 ± 0.11 _C_	2.96 ± 0.16 _A_	0.27 ± 0.11 _C_	0.62 ± 0.21 _BC_
2,5-Dihydroxybenzoic acid (mg/g DM)	nd	0.20 ± 0.01 _A_	0.16 ± 0.01 _AB_	0.15 ± 0.08 _AB_	0.07 ± 0.02 _B_
4-Hydroxybenzoic acid (mg/g DM)	nd	nd	nd	nd	nd
3-Hydroxybenzoic acid (mg/g DM)	nd	nd	nd	nd	nd
Vanillic acid (mg/g DM)	nd	nd	nd	nd	nd
Caffeic acid (mg/g DM)	0.09 ± 0.01 _B_	0.06 ± 0.01 _B_	0.23 ± 0.02 _A_	0.26 ± 0.01 _A_	0.09 ± 0.02 _B_
Syringic acid (mg/g DM)	nd	1.07 ± 0.24 _A_	nd	1.48 ± 0.31 _A_	0.43 ± 0.07 _B_
Vanillin (mg/g DM)	nd	nd	nd	nd	nd
*p*-Coumaric acid (mg/g DM)	0.02 ± 0.02 _B_	0.03 ± 0.01 _AB_	nd	0.02 ± 0.01 _B_	0.06 ± 0.02 _A_
Syringaldehyde (mg/g DM)	0.01 ± 0.01 _B_	0.01 ± 0.01 _B_	0.15 ± 0.02 _A_	0.16 ± 0.03 _A_	0.14 ± 0.02 _A_
Ferulic acid (mg/g DM)	0.02 ± 0.02 _A_	0.03 ± 0.01 _A_	0.04 ± 0.02 _A_	0.05 ± 0.02 _A_	0.02 ± 0.02 _A_
**Ferric Reducing Antioxidant Power (FRAP)**					
FRAP (g AAE/100 g DM)	5.40 ± 0.33 _B_	5.32 ± 0.35 _B_	5.71 ± 0.16 _AB_	6.38 ± 0.14 _A_	5.97 ± 0.04 _AB_
**DPPH Free Radical-Scavenging Activity**					
RSA_max_ (%)	89.4 ± 0.1 _C_	88.2 ± 0.2 _D_	91.5 ± 0.1 _A_	90.8 ± 0.3 _B_	90.4 ± 0.3 _B_
C RSA_max_ (mg/mL)	0.67 ± 0.02 _A_	0.67 ± 0.02 _A_	0.54 ± 0.02 _B_	0.54 ± 0.02 _B_	0.54 ± 0.02 _B_
IC50 (mg/mL)	0.24 ± 0.01 _A_	0.24 ± 0.01 _A_	0.22 ± 0.01 _B_	0.19 ± 0.01 _C_	0.19 ± 0.01 _C_

TPC: Total phenolic content; TFC: Total flavonoid content; TTC: Total tannin content; TPrC: Total proanthocyanidin content; THTC: Total hydrolyzable tannin content; RSA_max_: Maximum DPPH radical-scavenging activity; C RSA_max_: Concentration of a substance that provides maximum DPPH radical-scavenging activity; IC50: Concentration of a substance that provides 50% of DPPH radical-scavenging activity; nd: Not detected; GAE: Gallic acid equivalent; CE: Catechin equivalent; AAE: Ascorbic acid equivalent. Ascorbic acid (RSA_max_: 94.5 ± 0.2%; C RSA_max_: 0.05 ± 0.01 mg/mL; IC50: 0.02 ± 0.01 mg/mL); Catechin (RSA_max_: 88.7 ± 0.2%; C RSA_max_: 0.05 ± 0.01 mg/mL; IC50: 0.02 ± 0.01 mg/mL).

## Data Availability

The original contributions presented in this study are included in the article and Supplementary Materials. Further inquiries can be directed to the corresponding author.

## Supplementary Materials

The following supporting information can be downloaded at: https://www.mdpi.com/article/10.3390/antiox15020207/s1. Table S1: Estimated regression coefficients for phenolic recovery from CB by water-based CSE. Note: This model includes all the terms. Table S2: Estimated regression coefficients for phenolic recovery from CB by water–ethanol mixture CSE. Note: This model includes all the terms. Table S3: Analysis of variance for phenolic recovery from CB by water-based CSE. Note: This model includes all the terms. Table S4: Analysis of variance for phenolic recovery from CB by water–ethanol mixture CSE. Note: This model includes all the terms. Table S5: Estimated regression coefficients for phenolic recovery from CB by water-based ASE. Note: This model includes all the terms. Table S6: Estimated regression coefficients for phenolic recovery from CB by water–ethanol mixture ASE. Note: This model includes all the terms. Table S7: Estimated regression coefficients for the phenolic recovery from CB by water–methanol mixture ASE. Note: This model includes all the terms. Table S8: Analysis of variance for phenolic recovery from CB by water-based ASE. Note: This model includes all the terms. Table S9: Analysis of variance for phenolic recovery from CB by water–ethanol mixture ASE. Note: This model includes all the terms. Table S10: Analysis of variance for phenolic recovery from CB by water–methanol ASE. Note: This model includes all the terms.

## Abbreviations

The following abbreviations are used in this manuscript:

AAEs	Ascorbic acid equivalents
ANOVA	Analysis of variance
ASE	Accelerated solvent extraction
CB	Chestnut bur
CCRD	Central composite rotatable design
CEs	Catechin equivalents
CRSA_max_	The concentration of the extract that provides the maximum radical-scavenging activity
CSE	Conventional solvent extraction
DAD	Diode array detector
DM	Dry matter
DPPH	α,α-diphenyl-β-picrylhydrazyl
FRAP	Ferric reducing antioxidant power
GAEs	Gallic acid equivalents
HPLC	High-performance liquid chromatography
IC50	The concentration of the extract that provides 50% of the radical-scavenging activity
MAE	Microwave-assisted extraction
PLE	Pressurized liquid extraction
RSA	Radical-scavenging activity
RSA_max_	Maximum radical-scavenging activity
RSM	Response surface methodology
sc-CO_2_	Supercritical CO_2_ extraction
TFC	Total flavonoid content
THTC	Total hydrolyzable tannin content
TPC	Total phenolic content
TPrC	Total proanthocyanidin content
TTC	Total tannin content
UAE	Ultrasonic-assisted extraction
