# Supplementary material for: Accelerated Solvent Extraction as an Alternative for the Recovery of Phenolic Compounds from Chestnut Bur: Optimization of Extraction Conditions
Source: Antioxidants (Basel). 2026 Feb 4;15(2):207. doi: 10.3390/antiox15020207 (PMC12937611; doi:10.3390/antiox15020207)
Supplement: Supplementary file 1 [file antioxidants-15-00207-s001.zip › antioxidants-4087319-supplementary.pdf]

Supplementary material

Table S1. Estimated regression coefficients for phenolic recovery from CB by water-based CSE. Note: This model includes all the terms.

Term	Coefficient	Standard error coef.	T	p
Constant	5.68551	3.19978	1.777	0.119
T (°C)	-0.05008	0.07648	-0.655	0.533
t (h)	-0.89188	1.35122	-0.660	0.530
T (°C) * T (°C)	0.00007	0.00050	0.143	0.890
t (h) * t (h)	-0.79798	0.25218	-3.164	0.016
T (°C) * t (h)	0.04956	0.01522	3.257	0.014
S = 0.330329	PRESS=4.06545			
R-square = 86.90%	R-square (pred) = 30.28%	R-square (adj) = 77.55%		

Table S2. Estimated regression coefficients for phenolic recovery from CB by water-ethanol mixture CSE. Note: This model includes all the terms.

Term	Coefficient	Standard error coef.	T	p
Constant	1.55695	1.05378	1.477	0.170
Ethanol (%)	0.05343	0.00857	6.236	0.000
T (°C)	0.04573	0.02830	1.616	0.137
t (h)	0.41689	0.45265	0.921	0.379
Ethanol (%) * Ethanol (%)	-0.00098	0.00003	-28.073	0.000
T (°C) * T (°C)	-0.00015	0.00022	-0.685	0.509
t (h) * t (h)	-0.19974	0.08726	-2.289	0.045
Ethanol (%) * T (°C)	0.00007	0.00012	0.571	0.581
Ethanol (%) * t (h)	0.00132	0.00231	0.571	0.581
T (°C) * t (h)	0.00538	0.00578	0.932	0.374
S = 0.117661	PRESS=0.895991			
R-square = 99.57%	R-square (pred) = 97.25%	R-square (adj) = 99.19%		

Table S3. Analysis of variance for phenolic recovery from CB by water-based CSE. Note: This model includes all the terms.

Source	df	Sum of squares Seq.	Sum of squares Adjust.	Mean squares Adjust.	F	p
Regression	5	5.06750	5.06750	1.01350	9.29	0.005
Lineal	2	2.77606	0.07130	0.03565	0.33	0.732
T (°C)	1	2.37068	0.04680	0.04680	0.43	0.533
t (h)	1	0.40538	0.04754	0.04754	0.44	0.530
Quadratic	2	1.13414	1.13414	0.56707	5.20	0.041
T (°C) * T (°C)	1	0.04157	0.00222	0.00222	0.02	0.890
t (h) * t (h)	1	1.09257	1.09257	1.09257	10.01	0.016
Interaction	1	1.15730	1.15730	1.15730	10.61	0.014
T (°C) * t (h)	1	1.15730	1.15730	1.15730	10.61	0.014
Residual error	7	0.76382	0.76382	0.10912		
Lack of fit	3	0.52314	0.52314	0.17438	2.90	0.165
Pure error	4	0.24068	0.24068	0.06017		
Total	12	5.83132				

Table S4. Analysis of variance for phenolic recovery from CB by water-ethanol mixture CSE. Note: This model includes all the terms.

Source	df	Sum of squares Seq.	Sum of squares Adjust.	Mean squares Adjust.	F	p
Regression	9	32.3845	32.3845	3.5983	259.91	0.000
Lineal	3	21.4039	0.5404	0.1801	13.01	0.001
Ethanol (%)	1	18.1991	0.5384	0.5384	38.89	0.000
T (°C)	1	2.9965	0.0362	0.0362	2.61	0.137
t (h)	1	0.2082	0.0117	0.0117	0.85	0.379
Quadratic	3	10.9596	10.9596	3.6532	263.88	0.000
Ethanol (%) * Ethanol (%)	1	10.8839	10.9107	10.9107	788.11	0.000
T (°C) * T (°C)	1	0.0032	0.0065	0.0065	0.47	0.509
t (h) * t (h)	1	0.0725	0.0725	0.0725	5.24	0.045
Interaction	3	0.0210	0.0210	0.0070	0.51	0.686
Ethanol (%) * T (°C)	1	0.0045	0.0045	0.0045	0.33	0.581
Ethanol (%) * t (h)	1	0.0045	0.0045	0.0045	0.33	0.581
T (°C) * t (h)	1	0.0120	0.0120	0.0120	0.87	0.374
Residual error	10	0.1384	0.1384	0.0138		
Lack of fit	5	0.1011	0.1011	0.0202	2.71	0.149
Pure error	5	0.0373	0.0373	0.0075		
Total	19	32.5230				

Table S5. Estimated regression coefficients for phenolic recovery from CB by water-based ASE. Note: This model includes all the terms.

Term	Coefficient	Standard error coef.	T	p
Constant	-1.47340	0.398255	-3.700	0.008
T (°C)	0.08164	0.005695	14.336	0.000
t (min)	0.08932	0.020940	4.265	0.004
T (°C) * T (°C)	-0.00022	0.000023	-9.793	0.000
t (min) * t (min)	-0.00125	0.000452	-2.773	0.028
T (°C) * t (min)	-0.00028	0.000131	-2.116	0.072
S = 0.126641	PRESS=0.526672			
R-square = 99.18%	R-square (pred) = 96.15%	R-square (adj) = 98.59%		

Table S6. Estimated regression coefficients for phenolic recovery from CB by water-ethanol mixture ASE. Note: This model includes all the terms.

Term	Coefficient	Standard error coef.	T	p
Constant	1.19352	0.972687	1.227	0.248
Ethanol (%)	0.00103	0.013003	0.079	0.938
T (°C)	0.04136	0.011784	3.510	0.006
t (min)	0.11477	0.045416	2.527	0.030
Ethanol (%) * Ethanol (%)	-0.00073	0.000075	-9.752	0.000
T (°C) * T (°C)	-0.00005	0.000044	-1.125	0.287
t (min) * t (min)	-0.00195	0.000890	-2.186	0.054
Ethanol (%) * T (°C)	0.00022	0.000077	2.851	0.017
Ethanol (%) * t (min)	0.00048	0.000346	1.377	0.198
T (°C) * t (min)	-0.00052	0.000266	-1.949	0.080
S = 0.251159	PRESS=4.90866			
R-square = 98.70%	R-square (pred) = 89.89%	R-square (adj) = 97.53%		

Table S7. Estimated regression coefficients for the phenolic recovery from CB by water-methanol mixture ASE. Note: This model includes all the terms.

Term	Coefficient	Standard error coef.	T	p
Constant	-0.119420	1.04089	-0.115	0.911
Methanol (%)	0.021812	0.01391	1.569	0.148
T (°C)	0.041526	0.01260	3.297	0.008
t (min)	0.184824	0.04861	3.802	0.003
Methanol (%) * Methanol (%)	-0.000746	0.00008	-9.336	0.000
T (°C) * T (°C)	-0.000087	0.00005	-1.848	0.094
t (min) * t (min)	-0.002479	0.00095	-2.606	0.026
Methanol (%) * T (°C)	0.000354	0.00008	4.282	0.002
Methanol (%) * t (min)	-0.000714	0.00037	-1.924	0.083
T (°C) * t (min)	-0.000346	0.00029	-1.211	0.254
S = 0.268124	PRESS=5.03939			
R-square = 98.19%	R-square (pred) = 87.30%	R-square (adj) = 96.56%		

Table S8. Analysis of variance for phenolic recovery from CB by water-based ASE. Note: This model includes all the terms.

Source	df	Sum of squares Seq.	Sum of squares Adjust.	Mean squares Adjust.	F	p
Regression	5	13.5538	13.5538	2.7108	169.02	0.000
Lineal	2	11.9016	3.3006	1.6503	102.90	0.000
T (°C)	1	11.5902	3.2961	3.2961	205.52	0.000
t (min)	1	0.3114	0.2918	0.2918	18.19	0.004
Quadratic	2	1.5804	1.5804	0.7902	49.27	0.000
T (°C) * T (°C)	1	1.4571	1.5381	1.5381	95.90	0.000
t (min) * t (min)	1	0.1233	0.1233	0.1233	7.69	0.028
Interaction	1	0.0718	0.0718	0.0718	4.48	0.072
T (°C) * t (min)	1	0.0718	0.0718	0.0718	4.48	0.072
Residual error	7	0.1123	0.1123	0.0160		
Lack of fit	3	0.0615	0.0615	0.0205	1.62	0.319
Pure error	4	0.0507	0.0507	0.0127		
Total	12	13.6661				

Table S9. Analysis of variance for phenolic recovery from CB by water-ethanol mixture ASE. Note: This model includes all the terms.

Source	df	Sum of squares Seq.	Sum of squares Adjust.	Mean squares Adjust.	F	p
Regression	9	47.9262	47.9262	5.3251	84.42	0.000
Lineal	3	40.9600	1.0137	0.3379	5.36	0.019
Ethanol (%)	1	18.5844	0.0004	0.0004	0.01	0.938
T (°C)	1	22.0252	0.7770	0.7770	12.32	0.006
t (min)	1	0.3504	0.4029	0.4029	6.39	0.030
Quadratic	3	6.0942	6.0942	2.0314	32.20	0.000
Ethanol (%) * Ethanol (%)	1	5.7401	5.9996	5.9996	95.11	0.000
T (°C) * T (°C)	1	0.0526	0.0799	0.0799	1.27	0.287
t (min) * t (min)	1	0.3016	0.3016	0.3016	4.78	0.054
Interaction	3	0.8719	0.8719	0.2906	4.61	0.028
Ethanol (%) * T (°C)	1	0.5127	0.5127	0.5127	8.13	0.017
Ethanol (%) * t (min)	1	0.1196	0.1196	0.1196	1.90	0.198
T (°C) * t (min)	1	0.2396	0.2396	0.2396	3.80	0.080
Residual error	10	0.6308	0.6308	0.0631		
Lack of fit	5	0.5692	0.5692	0.1138	9.24	0.015
Pure error	5	0.0616	0.0616	0.0123		
Total	19	48.5570				

Table S10. Analysis of variance for phenolic recovery from CB by water-methanol ASE. Note: This model includes all the terms.

Source	df	Sum of squares Seq.	Sum of squares Adjust.	Mean squares Adjust.	F	p
Regression	9	38.9669	38.9669	4.3297	60.23	0.000
Lineal	3	30.7673	1.4245	0.4748	6.60	0.010
Methanol (%)	1	6.4829	0.1769	0.1769	2.46	0.148
T (°C)	1	23.2142	0.7815	0.7815	10.87	0.008
t (min)	1	1.0702	1.0392	1.0392	15.45	0.003
Quadratic	3	6.5098	6.5091	2.1697	30.18	0.000
Methanol (%) * Methanol (%)	1	5.8387	6.2658	6.2658	87.16	0.000
T (°C) * T (°C)	1	0.1828	0.2456	0.2456	3.42	0.094
t (min) * t (min)	1	0.4883	0.4882	0.4882	6.79	0.026
Interaction	3	1.6898	1.6898	0.5633	7.84	0.006
Methanol (%) * T (°C)	1	1.3183	1.3183	1.3183	18.34	0.002
Methanol (%) * t (min)	1	0.2661	0.2661	0.2661	3.70	0.083
T (°C) * t (min)	1	0.1054	0.1054	0.1054	1.47	0.254
Residual error	10	0.7189	0.7189	0.0719		
Lack of fit	5	0.6383	0.6383	0.1277	7.92	0.020
Pure error	5	0.0806	0.0806	0.0161		
Total	19	39.6858				
